# Does Medication-Related Osteonecrosis of the Jaw Influence the Quality of Life of Cancer Patients?

**DOI:** 10.3390/biomedicines8040095

**Published:** 2020-04-24

**Authors:** Gianluca Tenore, Ahmed Mohsen, Antonella Francesca Rossi, Gaspare Palaia, Federica Rocchetti, Andrea Cassoni, Valentino Valentini, Livia Ottolenghi, Antonella Polimeni, Umberto Romeo

**Affiliations:** Department of Oral Sciences and Maxillofacial Surgery, Sapienza University of Rome, 00161 Rome, Italy

**Keywords:** bisphosphonates, Medication-Related Osteonecrosis of the Jaw, osteonecrosis, quality of life, SF-12 questionnaire

## Abstract

The aim of this study is to observe the influence of Medication-Related Osteonecrosis of the Jaw (MRONJ) on the physical and mental conditions of cancer patients using a Quality of Life (QoL) questionnaire during regular dental practice measures. Twenty cancer patients (8 males and 12 females) with established MRONJ were enrolled in the “MoMax” (Oral Medicine and Maxillofacial) project of the Department of Oral Sciences and Maxillofacial Surgery at “Sapienza” University of Rome, and were included in the study. The 12-item Short Form Survey was used to evaluate the QoL. Statistical analysis revealed a significant difference for Mental Component Summary (MCS) scores based on age (*p* = 0.018). The regression analysis revealed that the Physical Component Summary (PCS) scores were negatively influenced by the anti-resorptive medication duration (*p* = 0.031 and β = −1.137). No significant differences were observed with the other variables considered. The QoL of cancer patients is generally deteriorated and MRONJ may cause a further negative impact. This study highlights the possible need to include psychosocial and physical evaluations in the management process of MRONJ in cancer patients.

## 1. Introduction

Medication-Related Osteonecrosis of the Jaw (MRONJ) is defined as an exposed or probed bone persisting for more than eight weeks in the maxillofacial region in patients with ongoing or a history of treatment with bone-modifying agents or angiogenic inhibitor agents [1,2,3]. Despite the incidence of MRONJ being relatively low, it should be considered as a potentially serious and debilitating complication [4].

The cumulative incidence of MRONJ ranges from 1% to 9% of patients with advanced cancer [3]. Although about 40% of patients with MRONJ are non-cancer patients, the epidemiological data show that the risk is higher in cancer patients, with a prevalence between 0.2% and 6.7%. In comparison, the prevalence is between 0% and 0.4% in patients with osteometabolic diseases [2,4]. This may be due to the existence of a high number of patients affected by osteometabolic diseases worldwide [4,5].

MRONJ, as one of the oral complications associated with cancer treatment, negatively affects the physical appearance, speech, breathing, and swallowing of patients. This negative impact correspondingly influences the Quality of Life (QoL) [6,7]. There is no totally approved treatment modality for MRONJ. Due to the nature of the disease and the general medical condition of this kind of patient, a palliative modality that decreases the symptoms has become one of the management modalities in many circumstances [8,9,10].

In cancer patients, traditional evaluations, such as measuring pain and symptoms for achieving the treatment goals, have become insufficient [11,12]. These kinds of evaluation do not provide the health provider with a comprehensive image of the patients’ psychosocial issues and all possible treatment benefits or side-effects. It may be inappropriate to consider relieving pain or survival as the goal of the treatment plan and neglect the physical and psychological status of the patients [12]. Additionally, it becomes a commitment for the dental team to tailor the dental management and prevention for cancer patients at risk of or with established MRONJ.

In order to confront the lack of information, a questionnaire for QoL evaluation may be a useful instrument for gathering general information about the patients’ physical and psychological conditions. In particular, the most recommended source of data of the patient for the assessment of QoL is the patient themself [11,13,14].

In a recent systemic review, the existence of few studies evaluating the QoL of MRONJ patients was observed. The authors recommended carrying out further studies on this topic and proposed developing and validating a specific QoL questionnaire for this complication, which may act as a guide for future treatment strategies and decisions [15].

The aim of this study is to observe the influence of MRONJ on the physical and mental conditions of cancer patients through the use of a QoL questionnaire during regular dental practice measures.

## 2. Experimental Section

A cross-sectional study was carried out among cancer patients that volunteered to enroll in the “MoMax” (Oral Medicine and Maxillofacial) project of the Department of Oral Sciences and Maxillofacial Surgery at “Sapienza” University of Rome; this project is a task force that was founded in June 2014 and designed to provide cancer patients and patients with Oral Potentially Malignant Lesions (OPML) with multidisciplinary team care. The main aim of this project is to customize and accelerate the treatment plan for cancer patients as a trial to improve their survival rate. Different health providers cooperate in this project, including oral medicine specialists, prosthodontists, maxillofacial surgeons, oncologists, radiotherapists, and histo-pathologists. All procedures performed in the study involving human participants were in accordance with the ethical standards of the institutional and/or national research committee and the 1964 Helsinki declaration and its later amendments or comparable ethical standards.

The inclusion criteria were patients with a confirmed diagnosis of cancer, current or a previous history of anti-resorptive or anti-angiogenic medications, established MRONJ with full clinical and radiographical investigations, and age ≥18 years old. Patients with the following criteria were excluded from the study: a history of radiotherapy, language difficulties, and mental disability.

Twenty-six patients fulfilling the inclusion criteria presented themselves to our department from September 2017 to April 2018. Twenty patients (8 males and 12 females) agreed to participate in the study and signed an informed consent form. Six patients were excluded from the study: five patients refused to participate and one patient was excluded due to language difficulties.

Full clinical and radiographical investigations, including Computed Tomography (CT), were carried out to achieve a definite diagnosis of MRONJ. The 12-item Short Form Survey (SF-12) version was used in this study. The SF-12 was administered in a face to face interview (including a health care worker and the patient) during their normal outpatient visit by the main researcher.

SF-12 is a shortened version of SF-36. It consists of 12 items that measure eight scales, including physical functioning (2 items), role physical (2 items), body pain (1 item), general health (1 item), vitality (1 item), social functioning (1 item), role emotional (2 items), and mental health (2 items). The response to scales differs across and within the same scale.

The standard scoring algorithms were used to produce the Physical and Mental Component Summary (PCS and MCS, respectively) scores of the SF-12 questionnaire. The PCS and MCS scores are the two recommended valuable aggregate summary measures. Low PCS and MCS scores indicate low level of the health status [16].

The recorded variables were the age, gender, marital status, tumor (cancer or cancer with bone metastasis), anti-resorptive medication timing (past or current), active principle of the drug, method of administration (intravenous (I.V), intramuscular (I.M), subcutaneous (S.C), oral, or association), anti-resorptive duration (<3 years or >3 years), number of infusions (<8 infusions or >8 infusions), MRONJ stage (according to the American Association of Oral and Maxillofacial Surgeons (AAOMS) staging system), and localization (mandible, maxilla, or both).

**Figure 1:**
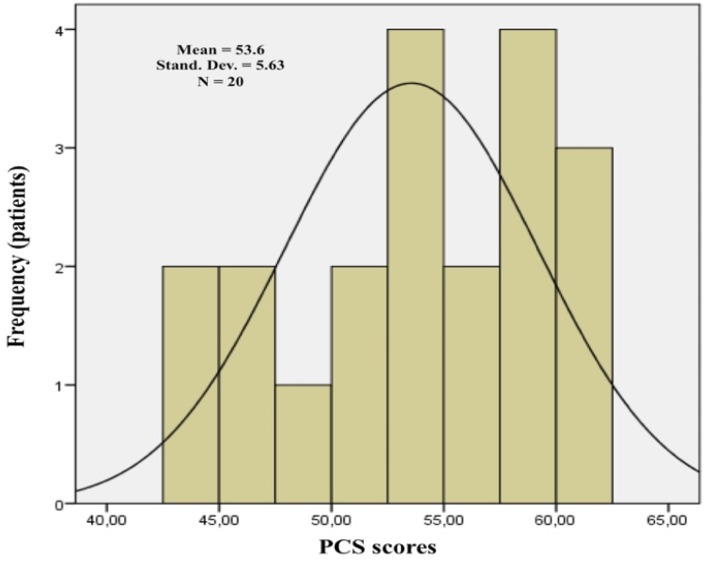
Physical Component Summary (PCS) histogram (created by SPSS software for Windows, release 20.0).

**Figure 2:**
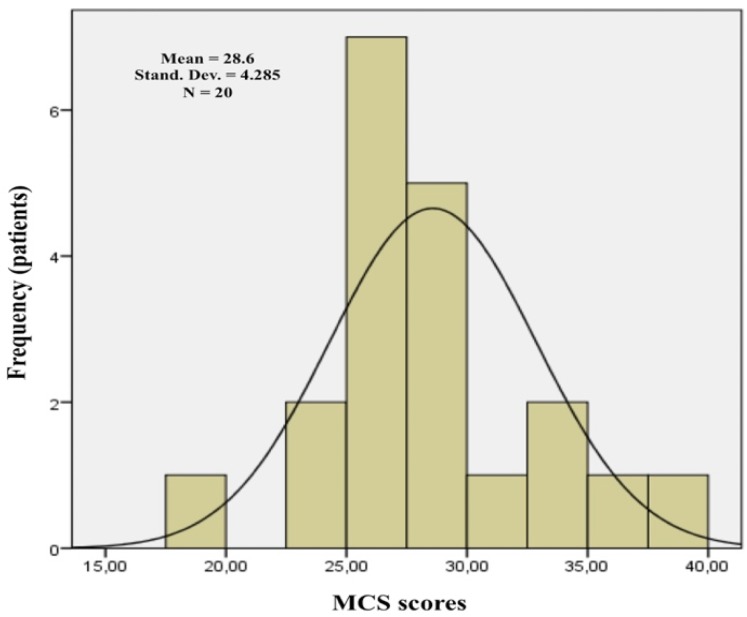
Mental Component Summary (MCS) histogram (created by SPSS software for Windows, release 20.0).

The data were analyzed statistically using the Statistical Package for the Social Sciences (SPSS) software for Windows, release 20.0. Mann–Whitney and Kruskal–Wallis tests were used for the quantitative variables to test the differences between groups. These non-parametric tests were chosen because the distribution of the various variable scores did not respect the assumptions of normality, as indicated by the skewness and kurtosis, and the Shapiro test. PCS and MCS scores were used as dependent variables for conducting the multiple linear regression analysis (Figure 1 and Figure 2). The significance level was set at *p* < 0.05.

## 3. Results

**Figure 3:**
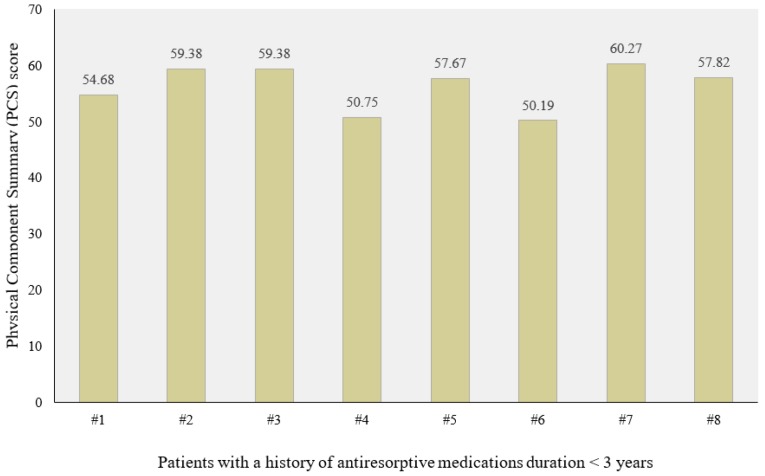
Physical Component Summary (PCS) in patients with a history of anti-resorptive medication duration <3 years.

**Figure 4:**
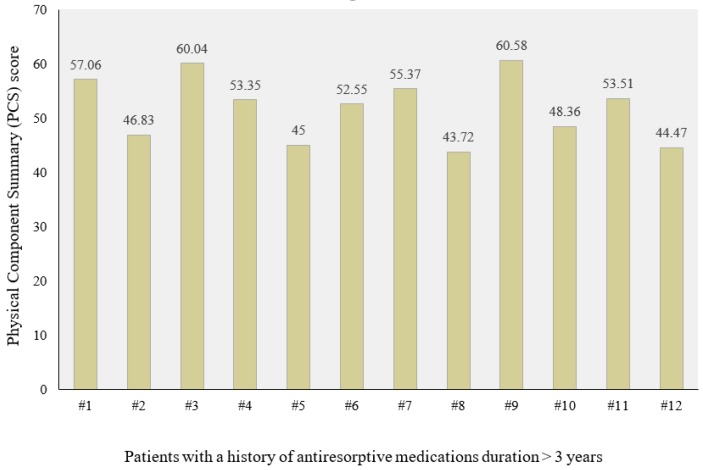
Physical Component Summary (PCS) in patients with a history of anti-resorptive medication duration >3 years.

Twenty patients with an average age of 66 years old were included. Six cases (30%) included established MRONJ at stage 0, 2 cases (10%) included that at stage I, 11 cases (55%) included that at stage II, and 1 case (5%) included that at stage III. The localization of MRONJ was distributed as follows: 3 cases were in the maxilla (15%), 12 cases were in the mandible (55%), and 5 cases were in both (Table 1). The Mann–Whitney test revealed a significant difference for MCS based on age. The group of older patients showed lower median scores than those of younger patients (*p* = 0.018). No further significant differences were observed between the groups considered (gender, marital status, type of tumor, medication timing, active principle, method of administration, duration of therapy, MRONJ stage, and localization) regarding either the PCS scores or the MCS scores (Table 2). It was observed, through the regression analysis, that the PCS scores were negatively influenced by the anti-resorptive medication duration. Patients who had been receiving treatment with anti-resorptive medications for less than three years showed higher PCS scores (*p* = 0.031 and β = −1.137). The other variables did not influence the dependent variables used (PCS and MCS) (Table 3, Figure 3 and Figure 4).

## 4. Discussion

MRONJ is a serious drug-related complication characterized by progressive destruction of the bone of the mandible and/or maxilla. It may lead to psycho-functional deterioration and eventually a poor QoL due to the possible occurrence of structural damage, pain, and suppurations [17]. Bone-modifying agents and angiogenic inhibitor agents are the causative drugs for MRONJ [3,13,16,18].

Although many pre-clinical and clinical studies on the pathogenesis of MRONJ have been carried out, the pathogenesis of the disease is not yet fully understood [19]. The collateral effects of drug administration, such as altered bone remodeling, the over-suppression of bone resorption, angiogenesis inhibition, the suppression of acquired immunity, vitamin D deficiency, and soft tissue bisphosphonate (BP) toxicity, have been suggested as an explanation of the unique localization of MRONJ. In addition, many co-factors, such as comorbidities (e.g., diabetes), smoking, dental interventions, and concurrent medications (e.g., corticosteroids), have been reported to play a role in the pathogenesis of MRONJ [19].

In a recent systemic review, it was found that the reported risk factors of this complication are missing uniformity. Some studies have only focused on the dental risk factors and others have only reported medical comorbidity risk factors [10]. The most commonly reported dental risk factor is dental extraction, while the most common medical comorbidity risk factor is chemotherapy (including hormone therapy and immunosuppressive therapy). Periodontal diseases and corticosteroid administration are the second most common reported risk factors [10].

A major risk for the development of MRONJ has been observed with I.V. bisphosphonates (BPs). Furthermore, oral BPs have been reported as a cause of MRONJ, but with a lower risk when compared to monthly I.V. BPs [1,2,20,21].

Numerous treatment protocols have been proposed, such as early conservative surgical approaches, extensive and radical surgical resections, and nonsurgical palliative protocols with the administration of long-term antibiotics [22,23]. However, there is no general approval for a specific intervention protocol. Although it has been demonstrated that some approaches have shown successful wound closure after the intervention, some reports have noted that these approaches may also cause worsening of the disease [2,9,22,23].

Some authors have suggested that success in MRONJ management can be considered in the case of the reduction of symptoms achieved by pain relief, infection control, and the prevention of further progression [9]. Consequently, many palliative protocols have been suggested as a trial to control this chronic complication, including hyperbaric oxygen, Photobiomodulation (PBM), and long-term antibiotics [8,9,23].

The involvement of a multidisciplinary team is an appropriate way of providing health care to cancer patients. A considerable impact on the clinical decision can be obtained by working as a team [24,25,26,27]. The nature of the disease, the absence of a fully approved treating modality, and the patient’s general medical condition have guided us in providing this kind of patient with multidisciplinary team care. Recently, the Multinational Association of Supportive Care in Cancer, International Society of Oral Oncology, and American Society of Clinical Oncology (MASCC, ISOO, and ASCO, respectively) have proposed a clinical practice guideline for the management of MRONJ. In this guideline, they have recommended the coordination of care for patients with a risk of or established MRONJ through cooperation between dentists and oncologists in the form of multidisciplinary team care [3].

In our study, no correlation was observed between age and PCS scores. However, in the literature, the analysis of three large-scale population studies demonstrated that age was associated with the physical and mental health scores (PCS and MCS) of SF-12 questionnaire version one. With standard algorism scoring, a negative correlation between age and PCS scores and a positive correlation with MCS scores were noted [16,28].

The relation between QoL and MRONJ stages has been observed [15]. Worsening of the QoL was observed with stage II. Miksad et al., noticed that stage “I” showed a better QoL and was similar to the control group of patients with a history of bisphosphonates [29]. Capocci et al., demonstrated a significant difference in the physical and mental health scores between stages I and III [17].

In our study, no relation was observed between the MRONJ stages and the summary components considered. This may be due to the small number of cases and the unequal distribution of cases among stages of MRONJ. The majority of cases were stage II (55%) and only three cases (15%) were stages I and III. This also may be due to the presence of about 30% of cases with stage 0.

We decided to include cases with MRONJ stage 0, because the AAOMS considers stage 0 to be a valid disease category. This is because the AAOMS has noted the presence of many studies that have demonstrated the progress of up to 50% of MRONJ cases from stage 0 to stage I, II, or III.

According to the AAOMS staging system, stage 0 is considered in patients with no clinical necrotic bone, but with non-specific symptoms or clinical and radiographic findings [2].

Our study showed a possible relation between PCS and the anti-resorptive medication duration (> 3 years and < 3 years). However, it was observed that some cases with more than a three year duration of anti-resorptive medication showed higher physical health scores. This may be due to the nature of MRONJ, as there are many other risk factors that may play a role in worsening the patient’s condition, such as the method of administration, the type of administrated drug, comorbidities, the underlying pathology, other concurrent medications, or the cause of drug administration. In addition, these drugs are prescribed in many circumstances as prophylaxis for metastasis in cancer patients not due to the bad condition of the underlying pathology.

The SF-12 is a multi-item survey for measuring the general health domains, without being specific to any disease or treatment groups. It is a shorter version of the SF-36 questionnaire. The main objective of its development is to reproduce the SF-36 questionnaire PCS and MCS. Many studies have been carried out to validate SF-12 and many authors have found that it can successfully produce the PCS and MCS of SF-36 [30,31,32,33]. It can be applied for measuring the health status of the general population or clinical populations and also for comparing general populations and diseased groups [34].

The SF-12 consists of eight scales derived from 12 items, which contain a mixture of positively- and negatively-worded responses. The eight scales are physical functioning, role physical, body pain, general health, vitality, social functioning, role emotional, and mental health scales. The eight scales are derived from one or two items. Since a greater number items means better representation of the scales, only the summary measures (PCS and MCS) have been considered the most useful scores derived from SF-12 [27,34].

PCS and MCS can be calculated through a scoring algorithm and there are computerized scoring algorithms available for producing them in real time. For SF-36, the calculation is usually performed by summing up the item score of each scale, transforming the resulting scale score into a 0–100 range, standardizing the scale scores into z-scores, and multiplying them with factor scores derived from orthogonally rotated principal component analyses (PCA) from the American general population. All of these procedures are carried out separately for the eight scales. For the calculation of PCS, positive weights of the resulting products of physical scales and negative weights of mental scales are summed, and the same procedure is performed for MCS, but vice versa. Then, the two scores are computed as norm-based t-scores [16,34].

Despite the frequent use of SF-12 and its advantages, some concerns have been reported and should be considered in the interpretation of the obtained results. First, SF-12 cannot provide detailed subscales, only summary scales (PCS and MCS). Second, concerns have been reported in regard to the standard scoring algorithms of PCS and MCS. This is because the summary scores of SF-12 are based on assuming that physical and mental health is uncorrelated, despite the contribution of all 12 items in the calculation of both PCS and MCS. Third, the items with responses representing higher PCS scores negatively affect the mental health scores (lower MCS) and vice versa. These points may lead to a decrease in the validity of these summary scores and the outcome of decisions based on these scores may not be achieved [16,27,28].

One of the main advantages of SF-12 is that a person does not need particular experience to use it. Moreover, SF-12 can be administrated in different modes, such as static (paper), online, or interactive voice response modes. The administration of SF-12 can be performed through self-administration or interviewer administration. The preferred administration type is the face to face interview, as several studies have demonstrated a bias for low summary scores with self-administration when compared to the interviewer type. SF-12 is also characterized as having a minimal respondent burden. It was found that 2–3 min were needed to complete it, which was about one third of the time needed for the completion of SF-36, as an example [34].

Some limitations were observed in this study that should be considered in future studies. First, the number of patients should be increased. Second, a disease-free control group would be useful for precisely evaluating the QoL of cancer patients with MRONJ. Further studies should test the SF-12 and other QoL instruments to validate and develop a specific questionnaire for evaluating QoL in MRONJ patients. The inclusion of other risk factors may improve the quality of the study in the evaluation of QoL in MRONJ patients, such as comorbidities (e.g., diabetes), concurrent medications (e.g., corticosteroids), and smoking.

## 5. Conclusions

This study highlights the possible need for including psychosocial and physical evaluations in the management process of MRONJ in cancer patients. Further studies using other QoL instruments with a higher number of patients and a disease-free control group are needed to observe the effect of MRONJ on the QoL of cancer patients.

## Figures and Tables

**Table 1:** Study sample characteristics.

Characteristics	Distribution (%)
**Age**	
Under 60	4 (20%)
Over 60	16 (80%)
**Gender**	
Male	8 (40%)
Female	12 (60%)
**Marital status**	
Single	5 (25%)
Married	14 (70%)
**Tumor**	
Cancer	8 (40%)
Cancer with bone metastasis	12 (60%)
**Anti-resorptive medication timing**	
Past	15 (75%)
Current	5 (25%)
**Active principle**	
Zoledronic acid	9 (45%)
Clodronic acid	1 (5%)
Alendronic acid	1 (5%)
Denosumab (one dose every month)	3 (15%)
Adalimumab	1 (5%)
Combination	5 (25%)
**Method of administration**	
I.V	11 (55%)
I.M/S.C	5 (25%)
Oral	1 (5%)
Association	3 (15%)
**Anti-resorptive medications duration**	
<3 years	8 (40%)
>3 years	12 (60%)
I.V. < 8 infusions	2 (10%)
I.V. + 8 infusions	12 (60%)
**MRONJ stage**	
0	6 (30%)
I	2 (10%)
II	11 (55%)
III	1 (5%)
**Localization**	
Maxilla	3 (15%)
Mandible	12 (60%)
Both	5 (25%)

**Table 2:** Median 12-item Short Form Survey (SF-12) component scores by patients’ characteristic variables.

Variable	*n*	Score of PCS-12 Median (min–max)	*p*-Value	Score of MCS-12 Median (max–min)	*p*-Value
**All the sample**	20	53.6 (43.7–60.6)		28.6 (19.6–37.9)	
**Age**			0.06		0.018
Under 60	4	48.8 (44.5–53.4)		31.9 (29.9–35.2)	
Over 60	16	56.2 (43.7–60.6)		27.4 (19.6–37.97)	
**Gender**			0.70		0.22
Male	8	54.02 (43.7–60.3)		28.789 (26.2–37.97)	
Female	12	54.5 (44.5–60.6)		27.4 (19.6–35.2)	
**Marital status**			0.71		0.58
Single	5	54.7 (43.8–60.1)		27.5 (25.4–37.97)	
Married	14	54.5 (44.5–60.6)		27.7 (19.6–35.2)	
Not declared	1	53.4		30.8	
**Tumor**			0.28		0.68
Cancer	8	57.4 (50.2–60.1)		27.7 (24.4–29.7)	
Cancer with bone metastasis	12	52.96 (44.5–60.6)		28.6 (19.6–37.97)	
**Anti-resorptive medication timing**			0.86		0.73
Past	15	53.5 (43.7–60.6)		27.9 (19.6–37.97)	
Current	5	57.1 (45–60.1)		27.4 (25.4–35.2)	
**Active principle**			0.57		0.54
Zoledronic acid	9	54.7 (43.7–60.6)		27.5 (19.6–37.97)	
Clodronic acid	1	57.1		28.8	
Alendronic acid	1	60.1		25.4	
Denosumab (one dose every month)	3	57.8 (50.8–60.3)		27.9 (26.5–29.8)	
Adalimumab	1	53.5		23.6	
Combination	5	50.2 (45–59.4)		29.7 (26.3–35.2)	
**Method of administration**			0.38		0.38
I.V	11	53.4 (43.7–60.6)		27.5 (19.6–37.97)	
I.M/S.C	5	57.1 (50.8–60.3)		27.9 (23.6–29.8)	
Oral	1	60.1		25.4	
Association	3	52.6 (45–59.4)		32.99 (27.4–35.2)	
**Anti-resorptive medications duration**			0.25		0.45
<3 years	8	57.1 (50.2–60.3)		29.3 (26.6–37.97)	
>3 years	12	51.7 (47.7–60.6)		28.1 (19.6–35.2)	
I.V < 8 infusions	2	57.4 (55.4–59.4)		25.9 (24.4–27.4)	
I.V + 8 infusions	12	51.4 (43.7–60.6)		29.8 (19.6–37.97)	
**MRONJ stage**			0.85		0.15
0	6	53.4 (46.8–60.1)		27.5 (23.6–30.8)	
I	2	51.4 (45–57.7)		31.4 (27.5–35.2)	
II	11	55.4 (43.7–60.6)		27.4 (19.6–34.5)	
III	1	54.7		37.97	
**Localization**			0.47		1.00
Maxilla	3	54.1 (43.7–60.3)		27.438 (23.6–37.97)	
Mandible	12	57.8 (45–60.6)		27.9 (19.6–35.2)	
Both	5	54.3 (46.8–60.1)		28.2 (25.4–30.8)	

**Table 3:** Multiple linear regression analysis.

Variable	PCS-12		MCS-12	
	β	*p*-Value	β	*p*-Value
Age	−0.188	0.55	−0.123	0.77
Gender	0.632	0.15	−0.633	0.25
Marital status	0.320	0.37	−0.136	0.76
Anti-resorptive medication timing	−0.830	0.08	1.018	0.09
Anti-resorptive medications duration	−1.137	0.03	0.471	0.32
Number of infusions	0.715	0.10	−0.652	0.21
MRONJ stage	−0.007	0.98	0.410	0.31
Localization	0.729	0.09	−0.643	0.21
